# Comparison of Clinical Manifestations, Antimicrobial Susceptibility Patterns, and Mutations of Fluoroquinolone Target Genes between *Elizabethkingia meningoseptica* and *Elizabethkingia anophelis* Isolated in Taiwan

**DOI:** 10.3390/jcm7120538

**Published:** 2018-12-11

**Authors:** Jiun-Nong Lin, Chung-Hsu Lai, Chih-Hui Yang, Yi-Han Huang

**Affiliations:** 1 School of Medicine, College of Medicine, I-Shou University, Kaohsiung 824, Taiwan; laich6363@yahoo.com.tw (C.-H.L.); je091410show@hotmail.com (Y.-H.H.); 2 Division of Infectious Diseases, Department of Internal Medicine, E-Da Hospital, I-Shou University, Kaohsiung 824, Taiwan; 3 Department of Critical Care Medicine, E-Da Hospital, I-Shou University, Kaohsiung 824, Taiwan; 4 Department of Biological Science and Technology, Meiho University, Pingtung 912, Taiwan; puppylovefu@gmail.com

**Keywords:** *Elizabethkingia meningoseptica*, *Elizabethkingia anophelis*, fluoroquinolone, quinolone-resistance determining region, drug resistance

## Abstract

*Elizabethkingia meningoseptica* and *Elizabethkingia anophelis* are two major pathogens in the genus *Elizabethkingia*. Studies have revealed that *Elizabethkingia anophelis* is frequently misidentified as *E. meningoseptica*. Therefore, our aim was to explore the clinical and molecular differences between these two species. The database of a clinical microbiology laboratory in a university-affiliated hospital of Taiwan was searched to identify patients with *Elizabethkingia* infections between January 2005 and June 2018. Species were reidentified using 16S ribosomal RNA gene sequencing. Twenty *E. meningoseptica* and 72 *E. anophelis* samples were collected from consecutive patients. *E. meningoseptica* was significantly more frequently isolated from the cerebrospinal fluid than was *E. anophelis*. The most susceptible antibiotic for all *Elizabethkingia* isolates was minocycline (91.3%), followed by levofloxacin (52.2%), tigecycline (23.9%), and piperacillin tazobactam (23.9%). Compared with *E. anophelis*, *E. meningoseptica* was significantly less susceptible to piperacillin tazobactam, minocycline, and levofloxacin. Regarding nonsynonymous substitutions in the quinolone-resistance determining regions of DNA gyrase, six sites were recognized in *E. meningoseptica* and one site was recognized in *E. anophelis*. *E. meningoseptica* had a significantly higher rate of fluoroquinolone target gene mutations than did *E. anophelis*. Because of less susceptibility to multiple antibiotics than *E. anophelis*, empirical antimicrobial therapy of *E. meningoseptica* should be more rigorous.

## 1. Introduction

*Elizabethkingia*, which is frequently distributed in the natural environments of soil and water, is a genus of aerobic, gram-negative, non-spore-forming, nonmotile, and nonfermenting bacilli [1,2]. This genus has been sporadically reported to cause severe infections in humans, particularly in neonates and immunocompromised patients [3,4,5,6,7,8,9]. Presently, six species are included in the genus *Elizabethkingia*, namely *E. meningoseptica*, *E. miricola*, *E. anophelis*, *E. bruuniana*, *E. ursingii*, and *E. occulta* [3]. Among these species, *E. meningoseptica* is the most well known [4]. However, *E. anophelis* has been increasingly reported to cause outbreaks in Singapore [5], Hong Kong [6], Taiwan [7], and the United States [8,9]. Other species are rarely reported to cause human infections.

Studies have demonstrated that some species of *Elizabethkingia* could not be accurately identified using traditional biochemical-based phenotyping methods or matrix-assisted laser desorption ionization–time of flight mass spectrometry (MALDI–TOF MS) platforms with a standard reference spectrum database [6,10]. In these microbial identification platforms, *E. meningoseptica* could be correctly identified. Nevertheless, *E. anophelis* is not included in the identification database, and this species is usually misidentified as *E. meningoseptica* by these machines [6,10]. The accurate identification of *E. anophelis* species relies on 16S ribosomal RNA (rRNA) gene sequencing or MALDI–TOF platforms with a “Research Use Only” database [6,8,10,11]. However, most studies investigating *Elizabethkingia* have used unreliable microbial identification methods; consequently, these studies actually presented the clinical or molecular characteristics of all *Elizabethkingia* species rather than those of each species.

In this study, to precisely explore the genuine epidemiology, clinical characteristics, and antimicrobial susceptibility patterns of *E. meningoseptica* and *E. anophelis*, we used 16S rRNA gene sequencing to accurately identify the species of *Elizabethkingia*. Subsequently, we compared *E. meningoseptica* and *E. anophelis*. Because fluoroquinolones were suggested to be an empirical antimicrobial therapy for *Elizabethkingia* infections [6], we also examined gene alteration in the quinolone-resistance determining regions (QRDRs) of *E. meningoseptica* and *E. anophelis* and investigated the association between QRDR mutations and fluoroquinolone resistance.

## 2. Materials and Methods

### 2.1. Study Setting and Design

This study was approved by the Institutional Review Board of E-Da Hospital (EMRP-106-105) and was conducted in accordance with the Declaration of Helsinki and national and institutional standards. The computer database of the clinical microbial laboratory in a 1000-bed university-affiliated medical center in Taiwan was searched for cultures that yielded *Elizabethkingia* species between January 2005 and June 2018. The API/ID32 Phenotyping Kits (bioMérieux, Marcy l’Etoile, France) and VITEK MS System (bioMérieux, Marcy l’Etoile, France) were used for microbial identification by the clinical microbial laboratory during 2005–2013 and 2014–2018, respectively. All isolates were stored as glycerol stocks at −80 °C until use. The species of *Elizabethkingia* was reidentified using 16S rRNA gene sequencing. Patients infected with *E. meningoseptica* and *E. anophelis* were included in this study. Clinical characteristics and outcomes were collected from the medical records. Inappropriate empirical antimicrobial therapy was defined as nonsusceptibility of the isolates to the empirically prescribed antibiotics. Shock was defined as systolic pressure of <90 mm Hg, a reduction of 40 mm Hg in systolic blood pressure from baseline, or a condition requiring inotropic agents to maintain blood pressure.

### 2.2. Species Identification Using 16S rRNA Gene Sequencing

After thawing, the frozen bacteria were subcultured on tryptic soy agar with 5% sheep blood (Becton Dickinson, Sparks, MD, USA). The colony was then inoculated on fresh tryptic soy agar with 5% sheep blood for overnight culture. The total DNA of fresh colonies was prepared using a Wizard Genomic DNA Purification Kit (Promega, Madison, WI, USA). The primers and protocols for amplification and sequencing of the 16S rRNA gene are listed in Supplementary Table S1 [7,10,12,13]. The assembled sequences of 16S rRNA were submitted to the National Center for Biotechnology Information website for comparison with their nucleotide sequences in GenBank sequence databases using the Basic Local Alignment Search Tool (https://blast.ncbi.nlm.nih.gov/Blast.cgi). The similarity of 16S rRNA sequences of isolates to the type strains in the GenBank sequence databases was examined (Supplementary Table S2). The species of *Elizabethkingia* were determined if the isolates shared ≥99.5% of 16S rRNA sequence identity with the type strains in GenBank [14]. The sequences of 16S rRNA were aligned using Clustal W with default options in MEGA7 [15]. Genetic relationships were inferred using the Maximum Likelihood method based on the JC69 model [15]. Phylogenetic tree was constructed in Dendroscope [16].

### 2.3. Antimicrobial Susceptibility Testing

The minimum inhibitory concentrations (MICs) of 18 antibiotics were determined using the broth microdilution method with Sensititre 96-well panels as per the manufacturer’s instructions (Thermo Fisher Scientific/Trek Diagnostics Systems, Oakwood Village, OH, USA). The susceptibilities were interpreted according to the criteria of “other non-*Enterobacteriaceae*” in the Clinical & Laboratory Standards Institute guidelines [17]. The MIC of tigecycline was interpreted according to the *Enterobacteriaceae* susceptibility breakpoints of the US Food and Drug Administration (susceptible MIC, ≤2 mg/L; intermediate MIC, 4 mg/L; resistant MIC, ≥8 mg/L) [18].

### 2.4. Identification of Mutations in the QRDRs

The primers and amplification conditions for QRDRs in *gyrA*, *gyrB*, *parC*, and *parE* of *E. meningoseptica* and *E. anophelis* are listed in Supplementary Table S1. The names and GenBank accession numbers of strains for comparison of QRDRs are listed in Supplementary Table S2.

### 2.5. Data Analysis

The data were analyzed using SPSS version 24.0 (IBM, Armonk, NY, USA). Categorical variables were calculated using the chi-squared test or Fisher exact test as appropriate. Variables associated with mortality were examined using univariate analyses. All variables were included in a logistic regression model of multivariate analysis with backward stepwise methods using the likelihood ratio. The significance of variables was calculated by odds ratios (ORs), 95% confidence intervals (CIs), and a two-tailed *p*-value. A *p*-value of <0.05 was considered statistically significant.

## 3. Results

### 3.1. Species Identification

**Figure 1:**
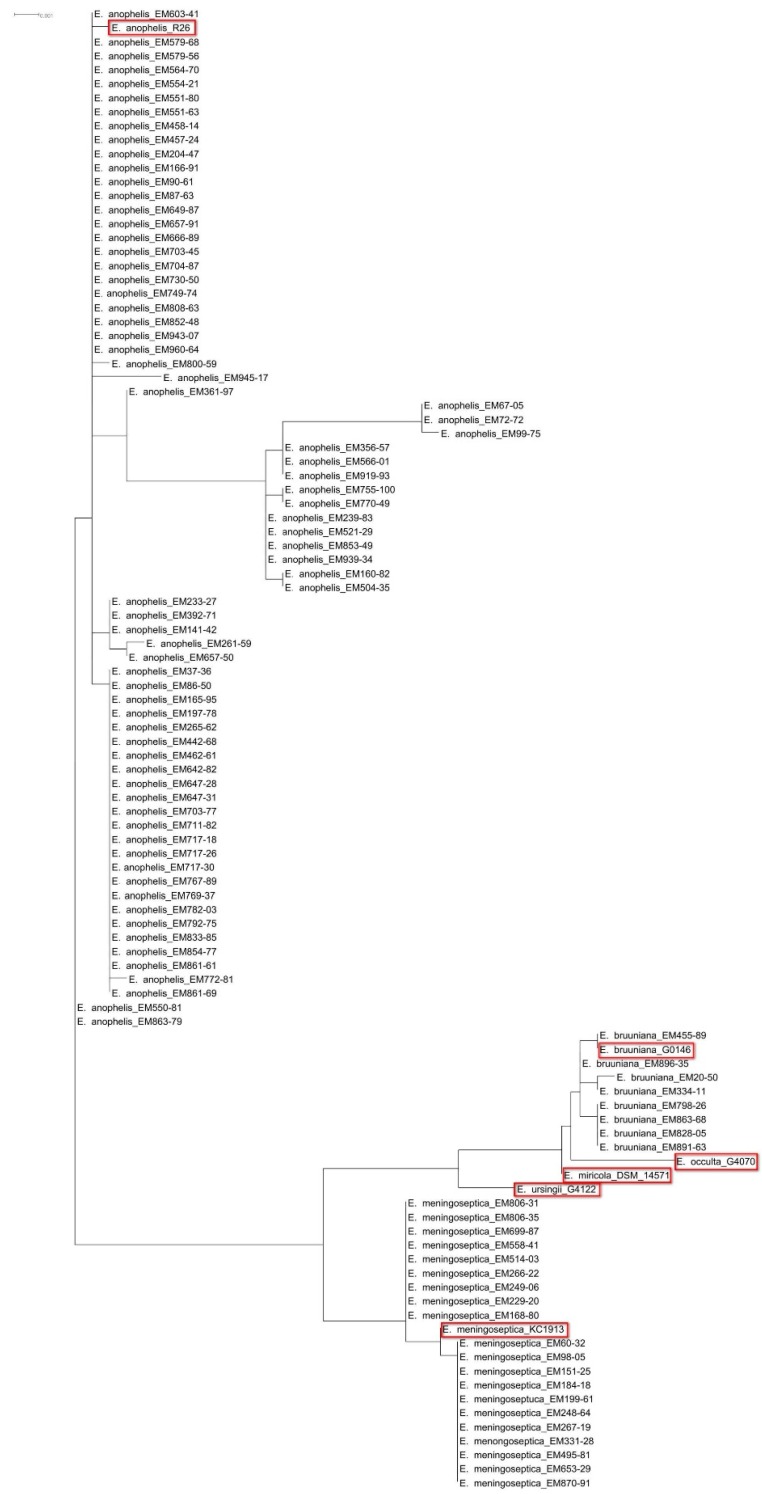
The phylogenetic tree based on 16S rRNA gene sequences among 20 *Elizabethkingia meningoseptica* isolates, 72 *Elizabethkingia anophelis* isolates, eight *Elizabethkingia bruuniana* isolates, and the six type strains of the *Elizabethkingia genus*. The tree is drawn to scale, with branch lengths in the same units as those of the evolutionary distances used to infer the phylogenetic tree. Red rectangles: the six type strains of the *Elizabethkingia* genus.

During the investigation period, 103 nonduplicated *Elizabethkingia* isolates were collected by the clinical microbiology laboratory. Three isolates died after thawing. After comparisons with the 16S rRNA gene sequences of type strains in GenBank (Supplementary Table S2), 20 isolates were identified as *E. meningoseptica*, 72 as *E. anophelis*, and eight as *E. bruuniana*. The 16S rRNA-based phylogenetic analysis clearly discriminated the species of these isolates (Figure 1).

### 3.2. Site of Isolation

Of these *E. meningoseptica* and *E. anophelis* isolates, the most common site of isolation was blood (60.9%), followed by the respiratory tract (12%), the tip of the central venous catheter (8.7%), bile (6.5%), and urine (4.3%) (Table 1). The other sites of isolation included cerebrospinal fluid (2.2%), ascites (2.2%), wounds/abscesses (2.2%), and pleural effusion (1.1%). Compared with *E. anophelis*, *E. meningoseptica* was significantly more frequently isolated from the cerebrospinal fluid (*p* = 0.045).

### 3.3. Clinical Characteristics of Elizabethkingia Infections

These 92 nonrepeated *Elizabethkingia* samples were isolated from 92 consecutive patients. Of these patients, men accounted for 69.6% and the median age was 61 years (Table 2). Chronic illness was found in 78.3% of the patients. The most common comorbidity was malignancy (43.5%), followed by hypertension (28.3%) and diabetes mellitus (26.1%). The majority of infections (89.1%) were attributed to healthcare-associated infection. Shock was recognized in 45.7% of the patients; and 47.8% of the patients were admitted to intensive care units. Empirical antibiotics included β-lactams (44.6%), β-lactam/lactamase inhibitors (21.7%), ciprofloxacin (10.9%), levofloxacin (26.1%), carbapenems (18.5%), aminoglycosides (9.8%), tigecycline (8.7%), and colistin (6.5%), either individually or in combination. Among these empirical antibiotic treatments, 80.4% were considered inappropriate empirical antimicrobial therapies. The overall case fatality rate of *Elizabethkingia* infections was 27.2%.

When patients with *E. meningoseptica* and *E. anophelis* infections were compared, no statistical differences were observed in the onset of age, sex, comorbidity, laboratory data, or case-fatality rate. Levofloxacin was more commonly used in patients with *E. anophelis* infection (*p* = 0.015). Inappropriate empirical antimicrobial therapy was more frequently recognized in patients with *E. meningoseptica* infections than in those with *E. anophelis* infections (*p* = 0.01; Table 2).

### 3.4. Factors Associated with Mortality

Univariate analysis showed that patients with liver cirrhosis (*p* = 0.032) and inappropriate empirical antimicrobial therapy (*p* = 0.02) had a higher mortality rate. Multivariate logistic regression analysis revealed that inappropriate empirical antimicrobial therapy was an independent risk factor for mortality in patients with *Elizabethkingia* infections (adjusted OR, 12.45; 95% CI, 1.33–116.77; *p* = 0.027; Table 3).

### 3.5. Antimicrobial Susceptibility Testing

The MICs and susceptibilities of *Elizabethkingia* are shown in Table 4. Except for piperacillin and piperacillin tazobactam, high MIC values were detected in β-lactams, β-lactam/β-lactam inhibitors, carbapenems, aminoglycosides, tetracycline, and trimethoprim-sulfamethoxazole. The *Elizabethkingia* isolates were most susceptible to minocycline (91.3%), followed by levofloxacin (52.2%), tigecycline (23.9%), and piperacillin tazobactam (23.9%).

When the susceptibility of each species was compared, *E. meningoseptica* generally had higher MIC values to multiple antibiotics than did *E. anophelis*. The isolates of *E. meningoseptica* were most susceptible to minocycline (60%), levofloxacin (30%), piperacillin (15%), and tigecycline (15%). Regarding the isolates of *E. anophelis*, the most susceptible antibiotic was minocycline (100%), followed by levofloxacin (58.3%), piperacillin tazobactam (30.6%), tigecycline (26.4%), and piperacillin (19.4%). Compared with *E. anophelis*, *E. meningoseptica* exhibited significantly lower susceptible rates to piperacillin tazobactam (*p* = 0.02), minocycline (*p <* 0.001), and levofloxacin (*p* = 0.025). Moreover, six *E. meningoseptica* isolates were resistant to all tested antibiotics, but none *E. anophelis* was resistant to all antibiotics (*p* < 0.001; Table 5).

### 3.6. Mutations in the QRDRs

The nonsynonymous substitutions of amino acids in the QRDRs of *E. meningoseptica* and *E. anophelis* are shown in Table 6. Isoleucine at position 83 in GyrA (*p* < 0.001), serine at position 95 in GyrA (*p* = 0.037), arginine at position 452 in GyrB (*p* = 0.044), and glutamine at position 470 in GyrB (*p* = 0.037) were associated with levofloxacin nonsusceptibility. No nonsynonymous substitutions were observed in the QRDRs of ParC and ParE. Of the 20 *E. meningoseptica* isolates, four were found to have amino-acid alteration at position 83 (Ser83Ile) in the QRDR of GyrA. Of the 72 *E. anophelis* isolates, nine and three possessed Ser83Ile and Ser83Arg in GyrA, respectively. *E. meningoseptica* had an additional five nonsynonymous alteration sites in the QRDRs, comprising two in GyrA (positions 95 and 102) and three in GyrB (positions 425, 452, and 470). However, *E. anophelis* had no amino acid alterations at these sites. When the fluoroquinolone target gene mutations were compared, *E. meningoseptica* had a significantly higher frequency of amino acid alterations at positions 95 and 102 in GyrA and positions 425 and 470 in GyrB than did *E. anopheles*.

## 4. Discussion

In this study, we used 16S rRNA gene sequencing as a reference method to identify species of *Elizabethkingia* collected during the past 13.5 years. We found that *E. anophelis*, rather than *E. meningoseptica*, accounted for the majority of human infections in the genus of *Elizabethkingia*. Furthermore, we recognized the differences in antimicrobial susceptibility patterns and amino-acid alterations in the QRDRs between *E. meningoseptica* and *E. anophelis*.

Since its first isolation in 1959 by Elizabeth O. King [4], *E. meningoseptica* has been reported to cause meningitis, pneumonia, bacteremia, and neutropenic fever in humans [2,19,20,21,22,23]. *E. anophelis*, which was first isolated from the midgut of a mosquito in 2011 [24], has also been reported to cause similar infections to *E. meningoseptica* [5,6,7,8,9]. Bacteremia has been determined to be a frequent presentation of *Elizabethkingia* infections [6,7,8,9,19,20,21,22,23]. Our study also showed that blood was the most common isolation site of both *E. meningoseptica* and *E. anophelis*. Although meningitis caused by *E. anophelis* has been reported [25], our study demonstrated that *E. meningoseptica*, as its name indicates, was more frequently associated with meningitis than was *E. anophelis*.

Studies have shown that most patients with *Elizabethkingia* infections had chronic underlying illnesses, such as diabetes mellitus, cardiovascular disease, chronic renal disease, malignancy, and liver cirrhosis [6,7,8,9,19,20,21,22,23]. Our study presents a similar result. The case fatality rate of patients with *E. anophelis* infection has been approximately 24–34% in previous reports [6,7,8,9]. However, the actual mortality rate of patients with species-confirmed *E. meningoseptica* is unclear. In our study, the case-fatality rate of *E. meningoseptica* was 30%, which is similar to that of *E. anophelis* (26.4%). We further analyzed the factors affecting mortality. After adjusting the confounding variables in the multivariate analysis model, we noted that inadequate empirical antimicrobial therapy was an independent risk factor for mortality. Therefore, how to choose appropriate empirical antibiotics for patients with *Elizabethkingia* infections is paramount.

Limited information exists on the antimicrobial susceptibility patterns of *Elizabethkingia.* As in the notable recent outbreak, several studies have shown that *E. anophelis* was usually resistant to multiple antibiotics, including most β-lactams, β-lactam/lactamase inhibitors, carbapenems, and aminoglycoside, but was variably susceptible to piperacillin, piperacillin tazobactam, minocycline, tigecycline, fluoroquinolones, and trimethoprim-sulfamethoxazole [6,7,8,9,26]. However, no data exist on the antimicrobial susceptibility patterns of *E. meningoseptica* identified using reliable methods. In our study, all *E. anophelis* isolates were susceptible to minocycline, and 58.3% and 30.6% were susceptible to levofloxacin and piperacillin tazobactam, respectively. By contrast, only 60% of *E. meningoseptica* isolates were susceptible to minocycline, 30% were susceptible to levofloxacin, and 5% were susceptible to piperacillin tazobactam. *E. meningoseptica* exhibited a significantly higher nonsusceptible rate to these antibiotics than did *E. anophelis*. The differences in the antimicrobial susceptibility patterns between *E. meningoseptica* and *E. anophelis* have never been presented in the literature. These results suggest that the choice of empirical antimicrobial therapy for *E. meningoseptica* should differ from that for *E. anophelis*.

Despite being a recommended antimicrobial therapy for *Elizabethkingia* infections, our study revealed that numerous *Elizabethkingia* isolates, particularly *E. meningoseptica*, were not susceptible to fluoroquinolones. Among the three mechanisms of fluoroquinolone resistance, gene mutations in the QRDRs of *gyrA*, *gyrB*, *parC*, and *parE* are a crucial mechanism of fluoroquinolone resistance [27]. Our study demonstrated that replacement of serine with isoleucine at position 83 (Ser83Ile) in the QRDR of GyrA was recognized both in *E. meningoseptica* and *E. anophelis*. Except for this, five additional sites of nonsynonymous alterations in GyrA and GyrB were identified in *E. meningoseptica* but not in *E. anophelis*. The QRDR mutation rates were significantly higher in *E. meningoseptica* than in *E. anophelis*. These results are compatible with the fact that *E. meningoseptica* was less susceptible to fluoroquinolones than was *E. anophelis*.

## 5. Conclusions

Our study reveals that *E. anophelis* was the predominant human pathogen in the genus *Elizabethkingia*. Although both species shared similar clinical manifestations, *E. meningoseptica* and *E. anophelis* exhibited significantly different antimicrobial susceptibility patterns and possessed diverse mutations in the target gene of fluoroquinolones. Because *E. meningoseptica* exhibits less susceptibility to multiple antibiotics than does *E. anophelis*, the empirical antimicrobial therapy of *E. meningoseptica* should be more rigorous and improved to be guided by antimicrobial susceptibility testing.

## Figures and Tables

**Table 1:** The isolation sites of *E. meningoseptica* and *E. anophelis.*

Site of Isolation	All (*n* = 92)	Number of Episodes (%)		OR (95% CI)	*p-*Value
		*E. meningoseptica* (*n* = 20)	*E. anophelis* (*n* = 72)		
Cerebrospinal fluid	2 (2.2)	2 (10)	0		0.045
Pleural effusion	1 (1.1)	0	1 (1.4)		0.999
Respiratory tract	11 (12)	1 (5)	10 (13.9)	0.33 (0.04–2.72)	0.445
Ascites	2 (2.2)	0	2 (2.8)		0.999
Bile	6 (6.5)	1 (5)	5 (6.9)	0.71 (0.08–6.41)	0.999
Blood	56 (60.9)	14 (70)	42 (58.3)	1.67 (0.58–4.84)	0.441
Tip of central venous catheter	8 (8.7)	1 (5)	7 (9.7)	0.49 (0.06–4.22)	0.681
Urine	4 (4.3)	1 (5)	3 (4.2)	1.21 (0.12–12.3)	0.999
Wound/Abscess	2 (2.2)	0	2 (2.8)		0.999

Abbreviations: OR, odds ratio; CI, confidence interval.

**Table 2:** Demographic characteristics, clinical information, and outcome of patients with *E. meningoseptica* and *E. anophelis* infections.

Characteristics	All (*n* = 92)	Number of Episodes (%)		OR (95% CI)	*p-*Value
		*E. meningoseptica* (*n* = 20)	*E. anophelis* (*n* = 72)		
Sex					
Male	64 (69.6)	15 (75)	49 (68.1)	1.41 (0.46–4.35)	0.55
Female	28 (30.4)	5 (25)	23 (31.9)	0.71 (0.23–2.19)	0.55
Age					
Range (year)	3–89	18–80	3–89		
Median (year)	61	61	62.5		
Mean ± standard deviation (year)	61.1 ± 17	56.6 ± 15.6	62.4 ± 17.3		0.179
Comorbidity					
Diabetes mellitus	24 (26.1)	6 (30)	18 (25)	1.29 (0.43–3.84)	0.652
Hypertension	26 (28.3)	4 (20)	22 (30.6)	0.57 (0.17–1.9)	0.354
End-stage renal disease	5 (5.4)	1 (5)	4 (5.6)	0.9 (0.09–8.49)	0.999
Malignancy	40 (43.5)	8 (40)	32 (44.4)	0.83 (0.3-2.28)	0.723
Liver cirrhosis	8 (8.7)	3 (15)	5 (6.9)	2.37 (0.51–10.89)	0.365
Chronic obstructive pulmonary disease	9 (9.8)	0	9 (12.5)		0.197
Type of infection acquisition					
Community-acquired infection	10 (10.9)	20	63 (87.5)		0.197
Healthcare-associated infection	82 (89.1)	0	9 (12.5)		0.197
Laboratory data					
White blood cell count (cells/mm^3^)	13,281 ± 8740	13,353 ± 6687	13,261 ± 9271		0.967
Hemoglobin (g/dL)	10.1 ± 2.1	9.8 ± 2.4	10.1 ± 2.1		0.585
Platelet count (×1000 cells/mm^3^)	228,570 ± 131,056	216,550 ± 157,332	231,900 ± 123,846		0.69
Serum creatinine (mg/dL)	1.8 ± 1.7	1.6 ± 1.3	1.9 ± 1.8		0.584
Empirical antimicrobial therapy					
β-lactams	41 (44.6)	11 (55)	30 (41.7)	1.71 (0.63–4.64)	0.289
β-lactam/lactamase inhibitors	20 (21.7)	4 (20)	16 (22.2)	0.88 (0.26–2.99)	0.999
Ciprofloxacin	10 (10.9)	1 (5)	9 (12.5)	0.37 (0.04–3.1)	0.685
Levofloxacin	24 (26.1)	1 (5)	23 (31.9)	0.11 (0.01–0.89)	0.015
Carbapenems	17 (18.5)	4 (20)	13 (18.1)	1.14 (0.33–3.96)	0.999
Aminoglycosides	9 (9.8)	3 (15)	6 (8.3)	1.94 (0.44–8.57)	0.402
Tigecycline	8 (8.7)	2 (10)	6 (8.3)	1.22 (0.23–6.58)	0.999
Colistin	6 (6.5)	1 (5)	5 (6.9)	0.71 (0.08–6.41)	0.999
Inappropriate empirical antimicrobial therapy	74 (80.4)	20 (100)	54 (75)		0.01
Shock	42 (45.7)	9 (45)	33 (45.8)	0.97 (0.36–2.62)	0.999
Admission to intensive care unit	44 (47.8)	9 (45)	35 (48.6)	0.87 (0.32–2.34)	0.775
Case fatality	25 (27.2)	6 (30)	19 (26.4)	1.2 (0.4–3.56)	0.748

Abbreviations: OR, odds ratio; CI, confidence interval.

**Table 3:** Factors associated with mortality in patients with *Elizabethkingia* infections.

Factor	Outcome		Univariate Analysis		Multivariate Analysis	
	Died	Survived	OR (95% CI)	*p-*Value	OR (95% CI)	*p-*Value
All Isolates (*n* = 92)						
Species						
*E. meningoseptica*	6 (24)	14 (20.9)	1.2 (0.4–3.56)	0.748	1.76 (0.5–6.16)	0.38
*E. anophelis*	19 (76)	53 (79.1)		0.748	0.57 (0.16–2)	0.38
Age ≥65 years	12 (48)	29 (43.3)	1.21 (0.48–3.04)	0.686	2.89 (0.86–9.74)	0.087
Sex, male	20 (80)	44 (65.7)	2.09 (0.7–6.3)	0.184	2.99 (0.78–11.52)	0.112
Underlying disease						
Diabetes mellitus	8 (32)	16 (23.9)	1.5 (0.55–4.12)	0.43	1.56 (0.44–5.49)	0.491
Hypertension	4 (16)	22 (32.8)	0.39 (0.12–1.27)	0.111	0.27 (0.07–1.11)	0.069
End-stage renal disease	1 (4)	4 (6)	0.66 (0.07-6.17)	0.999	0.45 (0.03–6.77)	0.566
Malignancy	12 (48)	28 (41.8)	1.29 (0.51–3.24)	0.593	1.11 (0.35–3.47)	0.86
Liver cirrhosis	5 (20)	3 (4.5)	5.33 (1.17–24.31)	0.032	4.67 (0.9–24.3)	0.067
Chronic obstructive pulmonary disease	4 (16)	5 (7.5)	2.36 (0.58–9.62)	0.248	1.98 (0.28–18.87)	0.492
Inappropriate empirical antimicrobial therapy	24 (96)	50 (74.6)	8.16 (1.03–64.97)	0.02	12.45 (1.33–116.77)	0.027
*E. meningoseptica* (*n* = 20)						
Age ≥65 years	3 (50)	4 (28.6)	2.5 (0.35–18.04)	0.613		0.999
Sex, male	5 (83.3)	10 (71.4)	2 (0.17–22.95)	0.999		0.999
Underlying disease						
Diabetes mellitus	2 (33.3)	4 (28.6)	1.25 (0.16–9.77)	0.999	4.5 (0.31–65.23)	0.27
Hypertension	0	4 (28.6)		0.267		0.999
End-stage renal disease	0	1 (7.1)		0.999		0.999
Malignancy	2 (33.3)	6 (42.9)	0.67 (0.09–4.93)	0.999	0.68 (0.03–17.96)	0.814
Liver cirrhosis	2 (33.3)	1 (7.1)	6.5 (0.46–91.92)	0.202	5.11 (0.3–87.96)	0.261
Chronic obstructive pulmonary disease	0	0				
Inappropriate empirical antimicrobial therapy	6 (100)	0				
*E. anophelis* (*n* = 72)						
Age ≥65 years	9 (47.4)	25 (47.2)	1.01 (0.35–2.88)	0.988	1.81 (0.36–8.97)	0.47
Sex, male	15 (78.9)	34 (64.2)	2.1 (0.61–7.22)	0.235	2.09 (0.53–8.19)	0.291
Underlying disease						
Diabetes mellitus	6 (31.6)	12 (22.6)	1.58 (0.49–5.04)	0.539	1.44 (0.36–5.76)	0.606
Hypertension	4 (21.1)	18 (34)	0.52 (0.15–1.79)	0.295	0.36 (0.09–1.46)	0.153
End-stage renal disease	1 (5.3)	3 (5.7)	0.93 (0.09–9.48)	0.999	0.66 (0.04–10.92)	0.773
Malignancy	10 (52.6)	22 (41.5)	1.57 (0.55–4.49)	0.403	1.3 (0.34–4.98)	0.706
Liver cirrhosis	3 (15.8)	2 (3.8)	4.78 (0.73–31.19)	0.111	3.4 (0.51–22.5)	0.204
Chronic obstructive pulmonary disease	4 (21.1)	5 (9.4)	2.56 (0.61–10.77)	0.231	3.06 (0.59–15.9)	0.183
Inappropriate empirical antimicrobial therapy	18 (94.7)	36 (67.9)	8.5 (1.05–69.04)	0.021	8.5 (1.05–69.04)	0.045

Abbreviations: OR, odds ratio; CI, confidence interval.

**Table 4:** The minimum inhibitory concentration and susceptibility of *Elizabethkingia* in this study.

Characteristics	All *Elizabethkingia* (*n* = 92)			*E. meningoseptica* (*n* = 20)			*E. anophelis* (*n* = 72)			OR (95% CI) ^d^	*p-*Value ^d^
	MIC_50_^a^	MIC_90_^b^	S, *n* (%) ^c^	MIC_50_^a^	MIC_90_^b^	S, *n* (%) ^c^	MIC_50_^a^	MIC_90_^b^	S, *n* (%) ^c^		
Piperacillin	64	>64	17 (18.5)	64	>64	3 (15)	64	>64	14 (19.4)	0.73 (0.19–2.85)	0.756
Piperacillin tazobactam	128/4	>128/4	22 (23.9)	128/4	>128/4	1 (5)	64/4	>128/4	22 (30.6)	0.12 (0.02–0.95)	0.02
Ticarcillin-clavulanic acid	>64/2	>64/2	0	>64/2	>64/2	0	>64/2	>64/2	0		
Ceftazidime	>16	>16	0	>16	>16	0	>16	>16	0		
Cefepime	>32	>32	1 (2.2)	>32	>32	0	>32	>32	2 (2.8)		0.999
Ceftriaxone	>32	>32	0	>32	>32	0	>32	>32	0		
Aztreonam	>16	>16	0	>16	>16	0	>16	>16	0		
Imipenem	>8	>8	0	>8	>8	0	>8	>8	0		
Meropenem	>8	>8	0	>8	>8	0	>8	>8	0		
Gentamicin	>8	>8	0	>8	>8	0	>8	>8	0		
Tobramycin	>8	>8	0	>8	>8	0	>8	>8	0		
Amikacin	>32	>32	4 (4.3)	>32	>32	0	>32	>32	4 (5.6)		0.573
Tetracycline	>8	>8	0	>8	>8	0	>8	>8	0		
Minocycline	<1	4	84 (91.3)	2	4	12 (60)	<1	2	72 (100)		<0.001
Tigecycline	4	>8	22 (23.9)	4	>8	3 (15)	4	8	19 (26.4)	0.49 (0.13–1.87)	0.382
Ciprofloxacin	2	>2	9 (9.8)	>2	>2	2 (10)	2	>2	7 (9.7)	1.03 (0.2–5.4)	0.999
Levofloxacin	2	>8	48 (52.2)	8	>8	6 (30)	2	>8	42 (58.3)	0.31 (0.11–0.89)	0.025
Trimethoprim-sulfamethoxazole	>4/76	>4/76	11 (12)	>4/76	>4/76	2 (10)	>4/76	>4/76	9 (12.5)	0.78 (0.15–3.93)	0.999

Abbreviations: *S*, *susceptible*; OR, odds ratio; CI, confidence interval; ^a^ MIC_50_, minimum inhibitory *concentration* at which 50% of the isolates tested are inhibited; ^b^ MIC_90_, minimum inhibitory *concentration* at which 90% of the isolates tested are inhibited; ^c^ The number of susceptible isolates and susceptible rates; ^d^ Comparison for the susceptible rates between *E. meningoseptica* and *E. anophelis*.

**Table 5:** The number of susceptible antibiotics among the 18 tested antibiotics for *E. meningoseptica* and *E. anophelis* infections.

Number of Susceptible Antibiotics	All Isolates (*n* = 92)	Number of Episodes (%)		OR (95% CI)	*p-*Value
		*E. meningoseptica* (*n* = 20)	*E. anopheles* (*n* = 72)		
0	6 (6.5)	6 (30)	0		<0.001
1	22 (23.9)	5 (25)	17 (23.6)	0.93 (0.29–2.93)	0.009
2	28 (30.4)	5 (25)	23 (31.9)	1.41 (0.46–4.35)	0.55
3	19 (20.7)	3 (15)	16 (22.2)	1.62 (0.42–6.23)	0.755
4	7 (7.6)	0	7 (9.7)		0.34
5	5 (5.4)	1 (5)	4 (5.6)	1.12 (0.12–10.6)	0.999
6	3 (3.3)	0	3 (4.2)		0.999
7	2 (2.2)	0	2 (2.8)		0.999

Abbreviations: OR, odds ratio; CI, confidence interval.

**Table 6:** Amino acid alternations in the QRDRs of GyrA and GyrB and levofloxacin susceptibility in *E. meningoseptica* and *E. anophelis.*

Amino Acid	Susceptibility of Levofloxacin				No. of Episode (%)		
	Susceptible (*n* = 48)	Non-Susceptible (*n* = 44)	OR (95% CI)	*p-*Value	*E. meningoseptica* (*n* = 20)	*E. anopheles* (*n* = 72)	*p-*Value
Position 83 of GyrA							
Serine	48 (100)	28 (63.6)		<0.001	16 (80)	60 (83.3)	0.478
Isoleucine	0	13 (29.5)		<0.001	4 (20)	9 (12.5)	
Arginine	0	3 (6.8)		0.105	0	3 (4.2)	
Position 95 of GyrA							
Serine	5 (10.4)	12 (27.3)	0.31 (0.1–0.97)	0.037	17 (85)	72 (100)	0.009
Proline	1 (2.1)	2 (4.5)	0.45 (0.04–5.11)	0.605	3 (15)	0	
Position 102 of GyrA							
Lysine	3 (6.3)	9 (20.5)	0.26 (0.07–1.03)	0.055	12 (60)	72 (100)	<0.001
Glutamine	3 (6.3)	5 (11.4)	0.52 (0.12–2.32)	0.473	8 (40)	0	
Position 425 of GyrB							
Isoleucine	2 (4.2)	7 (15.9)	0.23 (0.05–1.17)	0.081	9 (45)	72 (100)	<0.001
Lysine	4 (8.3)	7 (15.9)	0.48 (0.13–1.77)	0.263	11(55)	0	
Position 452 of GyrB							
Arginine	6 (12.5)	13 (29.5)	0.341 (0.12–0.996)	0.044	19 (95)	72 (100)	0.217
Serine	0	1 (2.3)		0.478	1 (5)	0	
Position 470 of GyrB							
Glutamate	5 (10.4)	12 (27.3)	0.31 (0.1–0.97)	0.037	17 (85)	72 (100)	0.009
Aspartate	1 (2.1)	2 (4.5)	0.45 (0.04-5.11)	0.605	3 (15)	0	

Abbreviations: QRDR, quinolone-resistance determining region; OR, odds ratio; CI, confidence interval.

## Supplementary Materials

The following are available online at http://www.mdpi.com/2077-0383/7/12/538/s1, Table S1: Primers and PCR conditions used in this study, Table S2: Information of strains for comparison of 16S rRNA and QRDRs in *gyrA*, *gyrB*, *parC*, and *parE* in this study.
