# Correction: Jiun-Nong Lin; Chung-Hsu Lai; Chih-Hui Yang and Yi-Han Huang. Comparison of Clinical Manifestations, Antimicrobial Susceptibility Patterns, and Mutations of Fluoroquinolone Target Genes between *Elizabethkingia meningoseptica* and *Elizabethkingia anophelis* Isolated in Taiwan. *Journal of Clinical Medicine* 2018, *7*, 538

**DOI:** 10.3390/jcm8040546

**Published:** 2019-04-22

**Authors:** Jiun-Nong Lin, Chung-Hsu Lai, Chih-Hui Yang, Yi-Han Huang

**Affiliations:** 1 School of Medicine, College of Medicine, I-Shou University, Kaohsiung 824, Taiwan; laich6363@yahoo.com.tw (C.-H.L.); je091410show@hotmail.com (Y.-H.H.); 2 Division of Infectious Diseases, Department of Internal Medicine, E-Da Hospital, I-Shou University, Kaohsiung 824, Taiwan; 3 Department of Critical Care Medicine, E-Da Hospital, I-Shou University, Kaohsiung 824, Taiwan; 4 Department of Biological Science and Technology, Meiho University, Pingtung 912, Taiwan; puppylovefu@gmail.com

The authors wish to make the following corrections to this paper [1].

The authors have made a critical typing error and a counting error in Table 2 as the parameters of “Community-acquired infection” and “Healthcare-associated infection” were mistakenly switched with each other.

Accordingly, Table 2, which is shown as follows:

**Table 2:** Demographic characteristics, clinical information, and outcome of patients with *E. meningoseptica* and *E. anophelis* infections.

Characteristics	All (*n* = 92)	Number of Episodes (%)		OR (95% CI)	*p-*Value
		*E. meningoseptica* (*n* = 20)	*E. anophelis* (*n* = 72)		
Sex					
Male	64 (69.6)	15 (75)	49 (68.1)	1.41 (0.46–4.35)	0.55
Female	28 (30.4)	5 (25)	23 (31.9)	0.71 (0.23–2.19)	0.55
Age					
Range (year)	3–89	18–80	3–89		
Median (year)	61	61	62.5		
Mean ± standard deviation (year)	61.1 ± 17	56.6 ± 15.6	62.4 ± 17.3		0.179
Comorbidity					
Diabetes mellitus	24 (26.1)	6 (30)	18 (25)	1.29 (0.43–3.84)	0.652
Hypertension	26 (28.3)	4 (20)	22 (30.6)	0.57 (0.17–1.9)	0.354
End-stage renal disease	5 (5.4)	1 (5)	4 (5.6)	0.9 (0.09–8.49)	0.999
Malignancy	40 (43.5)	8 (40)	32 (44.4)	0.83 (0.3-2.28)	0.723
Liver cirrhosis	8 (8.7)	3 (15)	5 (6.9)	2.37 (0.51–10.89)	0.365
Chronic obstructive pulmonary disease	9 (9.8)	0	9 (12.5)		0.197
Type of infection acquisition					
Community-acquired infection	10 (10.9)	20	63 (87.5)		0.197
Healthcare-associated infection	82 (89.1)	0	9 (12.5)		0.197
Laboratory data					
White blood cell count (cells/mm^3^)	13,281 ± 8740	13,353 ± 6687	13,261 ± 9271		0.967
Hemoglobin (g/dL)	10.1 ± 2.1	9.8 ± 2.4	10.1 ± 2.1		0.585
Platelet count (×1000 cells/mm^3^)	228,570 ± 131,056	216,550 ± 157,332	231,900 ± 123,846		0.69
Serum creatinine (mg/dL)	1.8 ± 1.7	1.6 ± 1.3	1.9 ± 1.8		0.584
Empirical antimicrobial therapy					
β-lactams	41 (44.6)	11 (55)	30 (41.7)	1.71 (0.63–4.64)	0.289
β-lactam/lactamase inhibitors	20 (21.7)	4 (20)	16 (22.2)	0.88 (0.26–2.99)	0.999
Ciprofloxacin	10 (10.9)	1 (5)	9 (12.5)	0.37 (0.04–3.1)	0.685
Levofloxacin	24 (26.1)	1 (5)	23 (31.9)	0.11 (0.01–0.89)	0.015
Carbapenems	17 (18.5)	4 (20)	13 (18.1)	1.14 (0.33–3.96)	0.999
Aminoglycosides	9 (9.8)	3 (15)	6 (8.3)	1.94 (0.44–8.57)	0.402
Tigecycline	8 (8.7)	2 (10)	6 (8.3)	1.22 (0.23–6.58)	0.999
Colistin	6 (6.5)	1 (5)	5 (6.9)	0.71 (0.08–6.41)	0.999
Inappropriate empirical antimicrobial therapy	74 (80.4)	20 (100)	54 (75)		0.01
Shock	42 (45.7)	9 (45)	33 (45.8)	0.97 (0.36–2.62)	0.999
Admission to intensive care unit	44 (47.8)	9 (45)	35 (48.6)	0.87 (0.32–2.34)	0.775
Case fatality	25 (27.2)	6 (30)	19 (26.4)	1.2 (0.4–3.56)	0.748

Abbreviations: OR, odds ratio; CI, confidence interval.

should be replaced with the following:

**Table 2:** Demographic characteristics, clinical information, and outcome of patients with *E. meningoseptica* and *E. anophelis* infections.

Characteristics	All (*n* = 92)	Number of Episodes (%)		OR (95% CI)	*p-*Value
		*E. meningoseptica* (*n* = 20)	*E. anophelis* (*n* = 72)		
Sex					
Male	64 (69.6)	15 (75)	49 (68.1)	1.41 (0.46–4.35)	0.55
Female	28 (30.4)	5 (25)	23 (31.9)	0.71 (0.23–2.19)	0.55
Age					
Range (year)	3–89	18–80	3–89		
Median (year)	61	61	62.5		
Mean ± standard deviation (year)	61.1 ± 17	56.6 ± 15.6	62.4 ± 17.3		0.179
Comorbidity					
Diabetes mellitus	24 (26.1)	6 (30)	18 (25)	1.29 (0.43–3.84)	0.652
Hypertension	26 (28.3)	4 (20)	22 (30.6)	0.57 (0.17–1.9)	0.354
End-stage renal disease	5 (5.4)	1 (5)	4 (5.6)	0.9 (0.09–8.49)	0.999
Malignancy	40 (43.5)	8 (40)	32 (44.4)	0.83 (0.3-2.28)	0.723
Liver cirrhosis	8 (8.7)	3 (15)	5 (6.9)	2.37 (0.51–10.89)	0.365
Chronic obstructive pulmonary disease	9 (9.8)	0	9 (12.5)		0.197
Type of infection acquisition					
Community-acquired infection	9 (9.8)	0	9 (12.5)		0.197
Healthcare-associated infection	83 (90.2)	20	63 (87.5)		0.197
Laboratory data					
White blood cell count (cells/mm^3^)	13,281 ± 8740	13,353 ± 6687	13,261 ± 9271		0.967
Hemoglobin (g/dL)	10.1 ± 2.1	9.8 ± 2.4	10.1 ± 2.1		0.585
Platelet count (×1000 cells/mm^3^)	228,570 ± 131,056	216,550 ± 157,332	231,900 ± 123,846		0.69
Serum creatinine (mg/dL)	1.8 ± 1.7	1.6 ± 1.3	1.9 ± 1.8		0.584
Empirical antimicrobial therapy					
β-lactams	41 (44.6)	11 (55)	30 (41.7)	1.71 (0.63–4.64)	0.289
β-lactam/lactamase inhibitors	20 (21.7)	4 (20)	16 (22.2)	0.88 (0.26–2.99)	0.999
Ciprofloxacin	10 (10.9)	1 (5)	9 (12.5)	0.37 (0.04–3.1)	0.685
Levofloxacin	24 (26.1)	1 (5)	23 (31.9)	0.11 (0.01–0.89)	0.015
Carbapenems	17 (18.5)	4 (20)	13 (18.1)	1.14 (0.33–3.96)	0.999
Aminoglycosides	9 (9.8)	3 (15)	6 (8.3)	1.94 (0.44–8.57)	0.402
Tigecycline	8 (8.7)	2 (10)	6 (8.3)	1.22 (0.23–6.58)	0.999
Colistin	6 (6.5)	1 (5)	5 (6.9)	0.71 (0.08–6.41)	0.999
Inappropriate empirical antimicrobial therapy	74 (80.4)	20 (100)	54 (75)		0.01
Shock	42 (45.7)	9 (45)	33 (45.8)	0.97 (0.36–2.62)	0.999
Admission to intensive care unit	44 (47.8)	9 (45)	35 (48.6)	0.87 (0.32–2.34)	0.775
Case fatality	25 (27.2)	6 (30)	19 (26.4)	1.2 (0.4–3.56)	0.748

Abbreviations: OR, odds ratio; CI, confidence interval.

The authors apologize for any inconvenience caused to the readers by these changes.
