# Supplementary material for: Comparison of Clinical Manifestations, Antimicrobial Susceptibility Patterns, and Mutations of Fluoroquinolone Target Genes between Elizabethkingia meningoseptica and Elizabethkingia anophelis Isolated in Taiwan
Source: J Clin Med. 2018 Dec 11;7(12):538. doi: 10.3390/jcm7120538 (PMC6306790; doi:10.3390/jcm7120538)
Supplement: Supplementary file 1 [file jcm-07-00538-s001.pdf]

J. Clin. Med. **2018**, *7*, 538; doi:10.3390/jcm7120538 www.mdpi.com/journal/jcm

Comparison of Clinical Manifestations, Antimicrobial Susceptibility Patterns, and Mutations of Fluoroquinolone Target Genes between *Elizabethkingia meningoseptica* and *Elizabethkingia anophelis* Isolated in Taiwan

Table S1. Primers and PCR conditions used in this study.

Primer	Sequence (5' to 3')	Amplicon size (bp)	GenBank accession no. or reference
PCR of 16S rRNA			
8f	CACGGATCCAGACTTTGAT(C/T)(A/C)TGGCTCAG	1498	Reference 12
1512r	GTGAAGCTTACGG(C/T)TAGCTTGTTACGACTT		
Sequencing of 16S rRNA			
8f	CACGGATCCAGACTTTGAT(C/T)(A/C)TGGCTCAG		Reference 7,10,13
534r	ATTACCGCGGCTGCTGG		
534f	CCAGCAGCCGCGGTAAT		
968f	AACGCGAAGAACCTTAC		
1512r	GTGAAGCTTACGG(C/T)TAGCTTGTTACGACTT		
PCR and sequencing of QRDR for *E. meningoseptica*			
gyrA-f[a]	CGATGTCGGTTATTGTTTCC	754	This study

gyrA-r[a]	GCAGTTCTGGCAATCATTTC		Accession no.
gyrB-f[b]	AGCGCGATGATATTCCGGTT	780	NR_042267
gyrB-r[b]	CCACATCGGCATCGGTCATA		
parC-f[b]	GTCTCCGGACTTTACCAGGA	477	
parC-r[b]	TGTGGAAAGACCTACCCCAAT		
parE-f[b]	AAGAGCCTGAAACATCAGAAGC	700	
parE-r[b]	CCAATAACAACCGGTTCCAG		
PCR and sequencing of QRDR for *E. anophelis*			
gyrA-f[b]	GGTTATCGTGTCCAGAGCG	446	
gyrA-r[b]	CCGCAATACCGGAAGTACCA		
gyrB-f[b]	ATACGCACGAAGGAGGTACG	847	This study
gyrB-r[b]	CGCTCTTTCTCGTTCCATGC		Accession no.
parC-f[b]	TGGTTTCTGGATTATGCCTCTT	446	CP007547
parC-r[b]	CCTACACCAATCCCTTCTACTCC		
parE-f[b]	AAAGAGCCAGAAACATCAGAGG	819	
parE-r[b]	TTCTTTTCTTTCGATGTCCGTA		

Abbreviations: PCR, polymerase chain reaction; rRNA, ribosomal RNA; QRDR, quinolone-resistance determining region

[a]The conditions were as follow: an initial extended denaturation step of 94°C for 5 min, followed by 32 cycles of 30 s at 94°C, 30 s at 50°C, 1 min at 72°C, and a final 5 min at 72°C.

[b]The conditions were as follows: an initial extended denaturation step of 94°C for 5 min, followed by 30 cycles of 30 s at 94°C, 30 s at 55°C, 1 min at 72°C, and a final 5 min at 72°C.

Table S2. Information of strains for comparison of 16S rRNA and QRDRs in *gyrA*, *gyrB*, *parC*, and *parE* in this study.

**Organism**	**Strain**	**Isolation source**	**Host**	**Collection date**	**Geographic location**	**GenBank accession number**	**Release date**	**Modify date**
**16S rRNA of type strains**								
E. meningoseptica	KC1913	Cerebrospinal fluid	*Homo sapiens*	1949	Massachusetts, USA	CP014338	2017/03/09	2018/01/21
E. anophelis	R26	Host midgut	*Anopheles gambiae*	2006	Sweden	CP023401	2017/09/11	2017/09/20
E. miricola	DSM 14571	Condensation water	Unknown	1997	Russian Space station Mir	MH789417	2018/08/28	2018/08/28
E. bruuniana	G0146	Unknown	*Homo sapiens*	Unknown	England, United Kingdom	CP014337	2017/03/10	2017/03/13
E. ursingii	G4122	Soil	Unknown	1964	Odense, Denmark	MH789420	2016/01/21	2018/05/02
E. occulta	G4070	Host sputum	*Homo sapiens*	1977	Melbourne, Australia	MH789418	2017/03/08	2018/05/02
**QRDRs in *gyrA*, *gyrB*, *parC*, and *parE***								
E. meningoseptica	G4076	Unknown	*Homo sapiens*	Unknown	England, United Kingdom	CP016376	2017/03/06	2017/03/08
E. anophelis	NUHP1	Host sputum	*Homo sapiens*	2012	Singapore	CP007547	2014/08/27	2017/04/02

Abbreviations: rRNA, ribosomal RNA; QRDR, quinolone-resistance determining region.
